# Progresses towards safe and efficient gene therapy vectors

**DOI:** 10.18632/oncotarget.5169

**Published:** 2015-09-04

**Authors:** Sergiu Chira, Carlo S. Jackson, Iulian Oprea, Ferhat Ozturk, Michael S. Pepper, Iulia Diaconu, Cornelia Braicu, Lajos-Zsolt Raduly, George A. Calin, Ioana Berindan-Neagoe

**Affiliations:** ^1^ Research Center for Functional Genomics, Biomedicine and Translational Medicine, University of Medicine and Pharmacy “Iuliu Haţieganu”, Cluj Napoca, Romania; ^2^ Department of Immunology and Institute for Cellular and Molecular Medicine, Faculty of Health Sciences, University of Pretoria, Pretoria, South Africa; ^3^ Department of Oncology and Pathology, Cancer Center Karolinska, Karolinska Institutet and Karolinska University Hospital, Stockholm, Sweden; ^4^ Department of Molecular Biology and Genetics, Canik Başari University, Samsun, Turkey; ^5^ Department of Immunology, University of Medicine and Pharmacy “Iuliu Haţieganu”, Cluj Napoca, Romania; ^6^ Department of Functional Genomics and Experimental Pathology, Oncological Institute “Prof. Dr. Ion Chiricuţă”, Cluj Napoca, Romania; ^7^ BlueBird Bio, MA, USA; ^8^ Department of Experimental Therapeutics, The University of Texas MD Anderson Cancer Center, Houston, TX, USA; ^9^ Center for RNA Interference and Non-Coding RNAs, The University of Texas MD Anderson Cancer Center, Houston, TX, USA; ^10^ Department of Physiopathology, Faculty of Veterinary Medicine, University of Agricultural Science and Veterinary Medicine, Cluj Napoca, Romania

**Keywords:** gene therapy, non-viral vectors, viral vectors, hybrid vectors, AAVP

## Abstract

The emergence of genetic engineering at the beginning of the 1970′s opened the era of biomedical technologies, which aims to improve human health using genetic manipulation techniques in a clinical context. Gene therapy represents an innovating and appealing strategy for treatment of human diseases, which utilizes vehicles or vectors for delivering therapeutic genes into the patients' body. However, a few past unsuccessful events that negatively marked the beginning of gene therapy resulted in the need for further studies regarding the design and biology of gene therapy vectors, so that this innovating treatment approach can successfully move from bench to bedside. In this paper, we review the major gene delivery vectors and recent improvements made in their design meant to overcome the issues that commonly arise with the use of gene therapy vectors. At the end of the manuscript, we summarized the main advantages and disadvantages of common gene therapy vectors and we discuss possible future directions for potential therapeutic vectors.

## INTRODUCTION TO GENE THERAPY

The discovery of restriction enzymes at the beginning of the 1970s opened the era of genetic manipulation that would become a major research theme for many researchers. While genetic engineering was advancing throughout the 1980s, the concept of gene therapy was starting to take shape in the researchers' view as a viable alternative for treatment of human diseases. This would imply that the genetic basis of a disease could be corrected using a “vehicle” or vector to deliver a therapeutic gene into the patients' body. On the basis of delivering the therapeutic gene product, all gene therapy protocols can be subdivided in ‘*in vivo*” gene delivery and “*ex vivo*” gene delivery. In the first approach the therapeutic gene is directly introduced into the patients' body to target the affected cells, while in the second approach, insertion of the delivery vector harboring the therapeutic gene is made in laboratory and the transformed/transduced cells are introduced back into the patients' body.

Great enthusiasm was generated around this innovative approach, when in 1990s the first “*ex vivo*” clinical trial was a success for a four-years-old girl with an adenosine deaminase deficiency (ADA). Blood cells taken from her bone marrow were treated with a recombinant ADA-retroviral derived vector and re-injected back into her blood stream. Although the corrected cells did expressed the ADA gene, she still had to take medication commonly prescribed for ADA deficiency as only a part of her white blood cells produced ADA [1]. Unfortunately this success was followed by the death of 18 year old Jesse Gelsinger, the first patient who died as a direct result of a gene therapy treatment [2]. An *in vivo* approach was used to correct the ornithine transcarbamylase (OTC) deficiency he suffered from, by injecting a recombinant adenovirus harboring the OTC gene directly into his blood stream. Four days after treatment, he died of multiple organ failure, most probably as a result of a severe immune response to the virus vector [2]. Future clinical trials performed in France on ten children with X-linked severe combined immunodeficiency (SCID-X1) or the so called “bubble boy” syndrome used an “*ex vivo*” approach to deliver the healthy gene into the blood cells of the patients via a retroviral-derived vector. After 30 months, two of the children developed leukemia, raising safety concerns regarding this type of vectors [3].

Despite approximately 1800 gene therapy clinical trials that have been reported world-wide up to 2012 [4], only one product has been approved to be used in clinical applications. This product, named Glybera, is used for treatment of lipoprotein lipase deficiency, by means of a virus which delivers the functional copy of the gene into the patient muscle cells [5]. This point to the fact that further optimization studies are needed to address the efficiency and safety of these vectors, so that gene therapy can become a clinical reality and a broader range of human diseases can be treated.

In this paper we review the major types of delivery systems used for gene therapy applications, pointing to innovations made in their design meant to overcome some of the drawbacks that limit their use by the medical community. At the end of the manuscript, we discuss future directions for development of gene therapy vectors that may lead to better clinical outcomes.

## NON-VIRAL VECTORS

**Figure 1:**
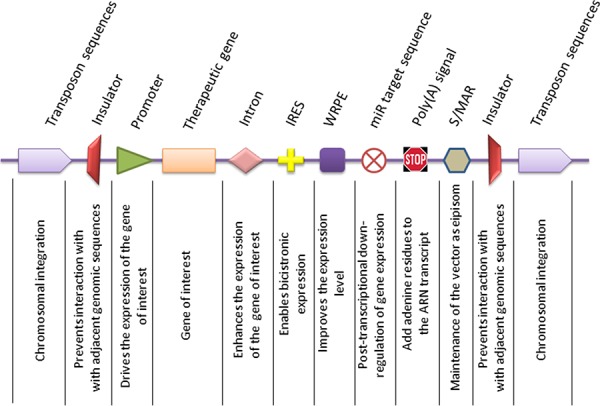
Representative components of gene delivery vectors Expression of the gene of interest or therapeutic gene is driven by an upstream promoter, either of exogenous or endogenous origin. Inclusion of an intron into the expression cassette assures higher transcription levels, as splicing and transcriptional are two coupled events. The internal ribosome entry site (IRES) permits co-transcription of two genes from the same transcript in a bicistronic manner. A further enhancement of gene expression can be achieved by using the Woodchuck Hepatitis Virus Posttranscriptional Regulatory Element (WPRE) to increase the level and stability of the nuclear transcripts. The expression of the therapeutic gene can be spatially limited to a specific cell type by inclusion of a miR recognition sequence at the 3′ end, which is recognized by its cognate miR transcript. In cells where the miR transcript is expressed, the activity of the therapeutic gene is suppressed, whereas in cells that are deficient in the specific miR, the expression of the therapeutic gene is de-repressed. The polyadenylation signal ensures properly sized transcripts. An optional element which can be included in the vector backbone is the scaffold matrix-associated region (S/MAR) which permits episomal replication and vector dilution in successive cell generations. An alternative to obtain stable and long term expression can be achieved by using transposon sequences for integration of the vector into the host genome. However, this implies the use of other genetic constituents, such as transposon trans-acting factors. In order to limit the activity of nearby genes, it would be desirable to flank the therapeutic expression cassette with insulator sequences.

These type of “vector” are comprised of synthetically produced biological particles, in which the plasmid DNA (pDNA) carrying the therapeutic gene expression cassette (Figure 1), is encapsulated or bound to a synthetic chemical compound and then released at the target site upon delivery. In contrast to viral-derived vectors, non-viral systems are relatively easy to produce, and the risk for inflammatory complications is lower [6]. Although the efficiency is reduced compared to viral vectors, non-viral vectors are of particular importance because besides pDNA, they are also capable of delivering synthetic compounds like oligonucleotides or siRNA [7].

**Figure 2:**
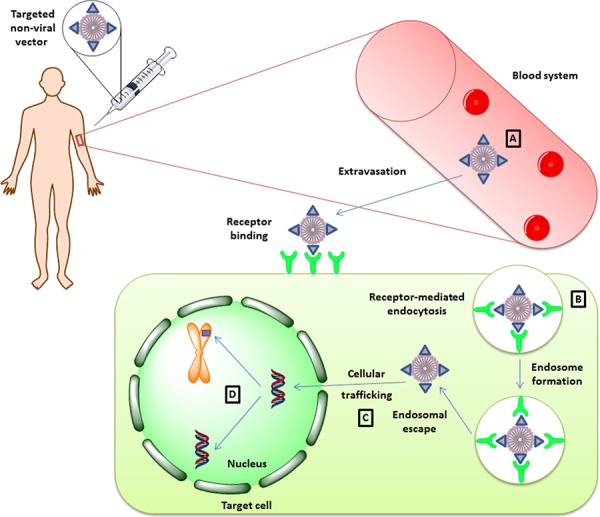
Therapeutic gene delivery mediated by non-viral vectors Successful gene delivery mediated by non-viral vectors encounters four major limitation steps. Once the vector is systemically administrated into the patient's blood stream **A.** it must preserve its integrity in order to be able to reach its target site in a functional state. After extravasation from the blood stream and migration into the extracellular stroma, the vector should be functionalized with a targeting peptide for interaction with the target cell in a receptor-dependent manner. Upon receptor binding, the vector particle is internalized as an endosomal vector **B.** Unless the vector escapes the endosome, it may be subjected to degradation, and this aspect can limit the transduction efficiency mediated by non-viral vectors. If the vector complex escapes endosomal degradation, it must migrate in an active manner in order to reach the nucleus and host transcription factors **C.** In the nucleus, the therapeutic genetic material can persist as an episome or it can integrate into the host genome, depending on the elements used in the construction of the vector **D.**

The limitations of non-viral vectors are related to extracellular stability of the delivery complex, internalization and the cellular trafficking of the vector, and the level and the sustainability of expression of the therapeutic gene (Figure 2).

### Extracellular stability of the delivery complex

This aspect implies that the non-viral vector once in the extracellular environment has to maintain its integrity in order to achieve physical contact with the target cell. Systemic administration of naked pDNA in the blood stream of mice proved to be inefficient, as the exogenous DNA is subjected to degradation by nucleases [8]. The remaining DNA accumulates in the liver [9] in non-parenchymal endothelial cells through “scavenger” receptors present on the surface of these cells [8] [10]. However, no detectable levels of transgene expression could be observed. In contrast, using a hydrodynamic injection method (intravenous administration of pDNA in a large volume of saline solution, at high pressure) significant expression of the transgene was seen in liver [10] due to an enlargement in the liver fenestrae and generation of membrane pores [11] or forced vesicular internalization [12]. However, because of these morphological changes in cell membranes, the hydrodynamic method is not feasible with human gene transfer.

These findings suggest that systemic gene delivery mediated by naked pDNA in humans is inefficient and that the therapeutic genetic payload needs to be attached to another compound which would increase its bioavailability and hence its efficiency. These compounds of synthetic origin are mainly represented by lipids or polymers, in which case the delivery complex is called lipoplex and polyplex, respectively (http://www.genetherapynet.com/non-viral-vectors/lipoplexes-and-polyplexes.html). Because the pDNA has a negative net charge, the chemical compounds are represented by cationic lipids in the case of lipoplex and cationic polymers in the case of polyplex. The use of cationic compounds would facilitate the electrostatic interaction of the gene delivery complex with the target cells and would also condense the genetic payload, protecting it from degradation (http://www.genetherapynet.com/non-viral-vectors/lipoplexes-and-polyplexes.html).

One of the standard delivery lipoplexes has been a simple pDNA/liposome conjugate, in which the positive charge of the lipid component condenses the pDNA. Among liposome formulations, those containing 3β-[N-(N',N'-dimethylaminoethane)carbamoyl]cholesterol (DC-Chol) and dioleoylphosphatidylethanolamine (DOPE) are the most efficient, and different molar ratios between the cationic lipid (DC-Chol) and helper lipid (DOPE) have been tested to improve the transfection efficiency [13]. However, such simple DNA/lipid formulations are rather preferred for local administration [14] than for systemic delivery. Under physiological conditions, DNA/liposome complexes tend to aggregate, limiting their circulation lifetime and hence their efficiency. Thus, further modifications have been made in the structure of cationic liposome, in order to overcome this limitation. A popular approach is to graft poly(ethylene glycol) (PEG) at the surface of the liposome, resulting in reduced aggregation of the delivery complexes and interaction with the plasma constituents [15] [16]. In other embodiments, solid lipid nanoparticles (SLNs) have been designed to overcome the limitations that commonly arise with the use of liposomes [17] [18].

Among the polyplexes, polyethylenimine (PEI) has been the “gold standard” of gene delivery via cationic polymers, because of its high transfection efficiency. However, the clinical utility of PEI-based-only complexes is limited due to their cytotoxic effects (http://hdl.handle.net/1721.1/62052). To circumvent this drawback, researchers have functionalized the PEI delivery complexes with different moieties like lipids [19], PEG [20] or pluronic polycarbamates [21] to improve their biological proprieties. Other research efforts have been directed to identify alternative cationic polymers to PEI. One such candidate is chitosan, a biocompatible polymer [22], but with a relatively lower transfection efficiency than PEI [23]. Grafting PEI onto chitosan nanoparticles proved to be a good strategy to improve the efficiency of such particles, without affecting their safety profile [24] [25]. Other polycationic alternatives like poly (β-amine esters) [26], cationic bolaamphiphile [27], dentrimers [28] or poly-D, L-succinimide [29] have also been proposed as delivery polyplexes with a lower cytotoxic profile.

### Internalization and cellular trafficking of the vector

The process of internalization of non-viral particles implies different aspects that include physical contact with the plasma membrane of the cell, endocytosis of the delivery complex, endosome release of the genetic payload, trafficking through the intracellular environment and nuclear import for the propose of therapeutic gene expression. In the case of small regulatory sequences like siRNA, these last two steps are not necessary because post-transcriptional mechanisms are targeted.

The distribution of the non-viral vectors is of major importance, as targeted transduction is a precondition of gene therapy. Systemic administration of liposome-based pDNA delivery complexes shows a rather unspecific distribution of gene expression, targeting different organs [30]. Such distribution would limit the delivery efficiency when the goal is to transduce a specific cell type. Therefore, receptor-specific ligands have been used to increase the specificity of non-viral vectors. These can range from natural ligands to peptides and antibodies (reviewed in [7]). For example, liposome-based vectors have been functionalized with hyaluronic acid (HA) to specifically transduce endothelial liver cells, because these cells express HA receptors [31]. Antigen-presenting cells have been shown to be efficiently transduced using a “mannosylated” PEI-based vector [32]. Chitosan-based vectors have also been subjected to modification that would imply attaching a functional entity, like a peptide, to increase their specificity and hence their efficiency [33].

Binding of the ligand to its cognate receptor determines endocytosis of the non-viral vector and the formation of endosome vesicles. At this point, the genetic payload needs to escape the endosome to avoid degradation. Polycations like PEI can act as “proton sponges” because of the amine groups can act as acceptors, leading to osmotic swelling and endosome disintegration, releasing the genetic payload into the cytoplasm. Because of PEI high toxicity, other potential cationic polymers with acceptor amine groups have been investigated as “safer” alternatives to PEI that could act as proton sponges [27] [29]. The proton acceptor property of a chlorochine analog, TP10, was used to enhance the efficiency of siRNA delivery, in which this compound was covalently attached to a cell-penetrating peptide, resulting in a peptide-based vector that would facilitate both internalization and endosome release of the payload [34]. A more recent study used a simplified means of internalization for delivering siRNA to the cytoplasm, by using a fusion protein composed of a cell-penetrating peptide and double-stranded RNA-binding domain, that can efficiently internalize its genetic-bound load by micropinocytosis (a type of fluid-phase endocytosis) or by direct penetration of the cell membrane [35].

Endosome-bypass could be an attractive approach for delivering therapeutic genetic material directly to the cytoplasm, simplifying the means of production of such vectors. The propriety of some viruses to escape the endosome degradation pathway could be exploited to design non-viral vectors. The hemaglutinating virus of Japan (HVJ) binds cell surface sialic receptors though its HN protein and the F protein mediates the fusion of the virus envelope with the cell membrane, delivering the viral genome into the cell. The relative ease with which exogenous genetic loads (pDNA, siRNA, miRNA, oligonucleotides) can be incorporated directly into inactivated HVJ constitutes an efficient way of generating non-viral vectors with enhanced efficiency for clinical applications (reviewed in [36]).

For cases in which post-transcriptional mechanisms are targeted, like in the case of siRNA delivery, endosome escape is sufficient for an therapeutic effect. However, in the case of expressing therapeutic genes, the DNA cargo, like pDNA, needs to migrate into the nucleus to access the host transcriptional machinery. Transport from the spot of endosome escape to the nucleus is a passive process which largely depends on the size of the plasmid, smaller plasmids been favored, as the cytoskeleton acts as a molecular sieve [37]. The delayed process of cellular trafficking can limit the efficiency of gene delivery, subjecting the genetic payload to the action of cytoplasmic nucleases. Again, the biology of some viruses has inspired researchers to improve non-viral vectors with functional entities that could facilitate nuclear import in an active manner. This has been the case in one study, in which the nuclear localization signal (NLS) of the Simian Virus (SV40) attached to the genetic payload via PNA (peptide nucleic acid) improved the efficiency of gene delivery to the nucleus up to 8-fold compared to vectors containing an inverted NLS sequence [38].

PNA is a DNA mimic, in which the negative deoxyribose phosphate backbone of DNA has been replaced by glycine, removing the negative charge of the molecule. This gives the possibility to co-synthetize PNA and peptides without complicated linking procedures. The technology, termed “bioplex”, is an innovative approach by which new functional entities can be attached to pDNA via hybridization (reviewed in [39]). Research efforts have been directed to improve the physical proprieties of the PNA-based delivery complexes [40]. This new concept of bioplex is a promising tool for development of future gene therapy vectors, because it gives the possibility to obtain gene delivery vehicles which could equal viruses in terms of efficiency, but without the safety concerns that virus-derived vectors impose, and the high costs and scaling up production drawbacks.

### The level and the sustainability of expression of the therapeutic gene

The level of therapeutic gene expression depends of the type of promoter used to drive its expression and this is directly correlated with the efficiency of gene transfer *in vivo*. However this efficiency also depends on the quantity of pDNA that is able to access the nucleus of the transfected cell, as discussed above.

Promoters used to drive gene expression can be classified depending on their origin into exogenous or viral promoters and endogenous or tissue promoters. The latter can further be subdivided into tissue-specific and non-tissue specific promoters. Initial findings have shown that although the use of tissue-specific promoters might be advantageous for targeted transcription (reviewed in [41]), their utility is limited due to low levels of gene transcription. However, later studies using tissue specific promoters, enhancers and introns substantially increased the long term expression up to therapeutic levels [42]. In contrast, viral promoters, like CMV, are known to drive initial very high levels of transcription, however they are subjected to the phenomenon of promoter inactivation in which cytokines and tumor necrosis factor TNF-α and interferon INF-γ are implicated, leading to transient gene expression [41]. Hybrid promoters composed of viral enhancer/endogenous fusions could be a preferred alternative to viral promoters, when prolonged expression of the therapeutic gene is desired [43]. Such fusions can enhance both the level and specificity of transgene expression [44].

Another aspect that can limit efficiency of gene expression is the ability of the immune system to recognize CpG unmethylated motifs of bacterial DNA, to which the immune cells react by releasing cytokines, resulting in inflammation. Indeed, eliminating the CpG motifs from bacterial genes expressed in mammalian systems can improve transgene expression levels [45]. The cytokines can produce an inflammatory response at the site of pDNA delivery, which can also result in repressing of therapeutic gene expression. Likewise, the number of CpG motifs in the promoter sequence can affect the level and sustainability of gene expression. Therefore choosing a CpG-free promoter in conjunction with a CpG-free plasmid backbone could make a difference to the success of the gene therapeutic effect. A list of CpG-free promoters with potential use for gene therapy applications has been reviewed elsewhere [46].

The use of tissue-specific promoters provides the possibility of controlling the expression of the therapeutic gene in a spatially-specific manner; however temporal control is also a key element in gene therapies. The tetracycline regulated system, which was originally described in *Escherichia coli*, is one of the best characterized and versatile types of system used for this propose. It is composed of two basic elements: the Tet repressor (TetR) and the tetracycline response element (*TRE*). By fusing the trans-activator domain of the HSV viral protein VP16 (a protein that recruits transcription factors), the TetR has been converted to the tTA trans-activator, and the tetracycline operator sequence (*tetO*) together with the minimal CMV promoter constitutes the *TRE*, which drives the gene expression. In the presence of tetracycline, the tTA is inactive, and the expression of the transgene is also repressed (Tet-Off). A mutant variant of tTA, called rtTA (reverse tTA) is capable of activating the transgene expression in the presence of doxycycline (Tet-On). Despite the fact that in the Tet-On system, the activity of the transgene is suppressed in the absence of doxycycline, there is basal “leakiness” of this system in the off state [47]. A recent study used the property of microRNA to silence gene expression to create a simpler and more versatile system, which could overcome some of the limitations seen in the Tet systems, *in vivo*. In this study, by adding four complementary sites of microRNA-122 at the 3′UTR of the vector, transgene expression was maintained at very low levels. The expression of the transgene could be restored repeatedly by using a microRNA antagonist, even 6 months after vector administration *in vivo* [48].

With respect to the sustainability of expression of the therapeutic gene, as discussed above, the type of promoter can have an impact on both the level and durability of gene expression. Even if long term expression is achieved by choosing an appropriate promoter, this is limited to non-dividing cells. In the case of cells which divide, the transgene-containing vectors are lost with each successive cell cycle. Therefore, other elements should be taken into account when designing vectors which are meant to transduce dividing cells. In order to maintain the vector in an episomal manner in the nucleus, two strategies have been investigated. One of these strategies exploits the potential of some viruses like simian virus (SV40), papilloma virus (HPV) or Epstein Barr virus (EBV) to replicate in the nucleus of the host cell as episomes. By incorporating viral *cis* replication elements and *trans*-acting protein coding sequences into the vector backbone, episomal plasmids are obtain that are able to maintain their presence in the cell lineage [49]. However, the expression of viral *trans*-acting proteins can surpass the expression of the therapeutic gene by activating the immune response against the cells expressing the viral proteins. This can limit the clinical applicability of such non-viral vectors that harbor viral sequences. The second approach to safely maintain the plasmid in an episomal manner is to include other elements that the immune system does not recognize as foreign molecules. One potential candidate is the scaffold/matrix associated region (S/MAR) of human interferon β, which mediates association of the plasmid-containing S/MAR to the chromosome scaffold via scaffold attachment factor-A (SAF-A). This interaction brings the plasmid DNA into the close proximity of the cell replicating machinery, favoring its replication with each successive cell cycle and its maintenance as an episomal entity for hundreds of cell generations [50] [51] [44].

Another way to obtain durable expression is to design vectors which integrate into the host genome. The capability of some bacteriophages to insert their genetic material into the host bacterial genome has been exploited by researchers to obtain non-viral vectors that would be able to stably integrate their therapeutic cargo into the target host genome. Bacteriophages in their lysogenic cycle integrate into the bacterial genome by a homologous recombination phenomenon, a process which is mediated by enzymes called site-specific recombinases (SSR). These enzymes recognize unique sites (RS) on the virus genome and the host genome, at which the recombination takes place. On the human genome “pseudosites” have been identified that could be recognized by SSR. Therefore several SSRs have been described, like Cre recombinase (RS-*lox*P), integrase γ (RS-*aat*P x *aat*B) and phiC31. As Cre recombinase can mediate the reverse excision phenomenon, and γ integrase requires bacterial factors, integrase phiC31 is a more appropriate candidate for stable insertion of the therapeutic gene. Although eleven RS have been identified on the human genome for phiC31, it remains to be determined whether integration at these sites could have a tumorigenic effect [52]. A recent study identified a locus on chromosome 8p22 (*DLC1*) as a candidate for gene therapy applications, which has not been associated with insertional mutagenesis upon integration of an exogenous transgene [53].

DNA transposons have attracted the interest of the biomedical community as useful genetic tools for delivering genes into mammalian genomes because of their capability of mobilization by a simple “cut and paste” mechanism. This class of mobile genetic elements is poorly represented in the human genome compared to their retro-transposon counterparts, and their activity subsided about 31 million years ago [54] [55]. A representative member of this class of transposable elements is the TC1/mariner transposon, which is composed of two terminal inverted repeats (TIRs) that flank the transposase gene [56]. The “resurrected” TC1/mariner transposon, called the “Sleeping Beauty” has been developed for stable insertion of foreign genes into mammalian genomes by random insertional transposition, and its clinical importance for treatment of human diseases is beginning to emerge [57] [Chénais, 2013 http://dx.doi.org/10.4236/ojgen.2013.32A1001]. Gene delivery mediated by the Sleeping Beauty transposon system comprises two plasmids, one that contain the transgene expression cassette flanked by two TIRs and a helper plasmid that encodes the transposase. Pre-clinical in *vitro* and in *vivo* experiments have already underlined the versatility of this gene transfer system for future clinical applications [58]. In addition to the Sleeping Beauty transposon, other emerging DNA-transposon-based systems, like PiggyBac transposon, have proven usefulness as safe and efficient platforms for gene therapy [59].

## VIRAL-DERIVED VECTORS

Viruses represent appealing tools for therapeutic gene transfer because of their high transfection/transduction efficiency in wide range of human cells. As viruses are pathogenic agents, they need to be attenuated to be safely used in clinical applications. In this regard, virus-derived vectors have been designed that originate from different viral classes like adenoviruses (Ad), adeno-associated viruses (AAV), retroviruses and lentiviruses. Beside these types, other virus categories have been investigated for gene transfer. Approximately 70% of the vectors used in gene therapy clinical trials are represented by viral-based delivery systems [4]. However, there are a few failures that negatively marked the past of gene therapy, which imply that further optimization is needed to safely use this type of vectors for future clinical proposes.

### Adenoviral vectors

Adenoviruses are a family of DNA viruses, which are comprised of a double stranded DNA genome of 36 kilobases (Kb) encapsulated within the viral capsid. Transduction of the host cell is initiated by binding of the coxsackievirus and adenovirus receptors (CAR) via the knob domain of the fiber protein of the viral capsid. This event is followed by interaction of the viral penton base with cell surface integrins, which results in the internalization of the virus via receptor-mediated endocytosis. Once in the cell, the virion escapes the endosome and the viral particle is disassembled, while the viral genome translocates to the nucleus, where it replicates in an episomal manner [60].

One way to design adenoviral vectors is to delete the viral genes that are responsible for replication, in which case the resulting vectors are replication-defective. When the viral genes are kept in their design, adenoviral vectors are replication-competent.

### Replication-defective vectors

Therapeutic gene delivery via adenoviral vectors implies that once the gene is delivered into the target cell, the virion must not enter its normal lysogenic life cycle. This would result in cell lysis and the expression of the transgene would therefore be compromised. One approach is to generate deletions in the E1 and E3 regions of the viral genome, which results in replication-defective viral particles. This type of vector, called first-generation adenoviral vectors (RAd), in which the transgene expression cassette is inserted in the E1 region, is dependent on a special cell line to provide *in trans* the E1 viral proteins. However, RAd still pose safety concerns because they can trigger an immune response towards the expressed viral proteins from the viral construct in the infected cells [47].

To overcome this limitation, high-capacity or helper-dependent (HD-Ad) adenoviral vectors have been designed, in which the entire viral gene set has been removed. This also gives the possibility to clone transgene expression cassettes up to 36 Kb. The HD-Ad vectors retain only the inverted terminal repeats (ITR) and the packaging signal (ψ) of the wild-type adenovirus. A helper virus is required to provide *in trans* the viral proteins necessary for assembly of the viral particles [61]. However, a major drawback of this system is contamination of the vector batch with helper viruses. In addition, recombination events between the vector and helper genomes are quite frequent, despite recent improvements in helper virus design [62] [63]. Such events could result in replication-competent HD-Ad vectors or helper viruses containing a packaging signal. Therefore, the titer of pure HD-Ad vectors is very low, which makes large-scale production inefficient.

The activation of the immune response upon systemic or local administration of adenoviral vectors has led to the implementation of strategies that would circumvent this limitation of therapeutic gene delivery using these vectors. One such strategy would be to change the adaptive immune response towards the adenoviral vectors. In one pre-clinical study, a dendritic cell-based strategy has been successfully used to induce tolerance to adenoviral vectors [64]. Other strategies have exploited the lack of pre-existent immune memory towards non-human adenoviruses, which would make such vectors attractive tools for gene therapy. The canine adenovirus type 2 (CAV-2) has gained attention as being one of the most highly characterized non-human adenoviruses with potential for clinical applications such as neurodegenerative disorders [65]. Production of such vector particles is dependent on canine cell lines to provide the viral proteins *in trans*, and efforts have been made to obtain clinical grade CAV-2 vectors [66] [67].

As mentioned above, transfection of host cells is dependent on the existence of CAR receptor expression at the cell surface. This propriety would limit the spectrum of cell types that are prone to infection with adenoviruses. Systemic administration of adenoviruses leads to preferential transduction of the liver cells [68], a result with potential cytotoxic effects. Some improvements have been made to reduce the host immune response to the vector by linking polymers to the virus vector capsid, such as PEG [69], PEI-CyD-FA [70] or PEG/PEI [71], which lead to reduced toxicity upon administration compared to non-modified adenoviral vectors. Liposome-coated adenoviral vectors also show promise to increase the biosafety profile of these vectors. In addition, such vector formulation retargeted the viral-driven expression of a transgene from liver to the lung, a result with potential applications for gene therapy of lung diseases [72].

**Figure 3:**
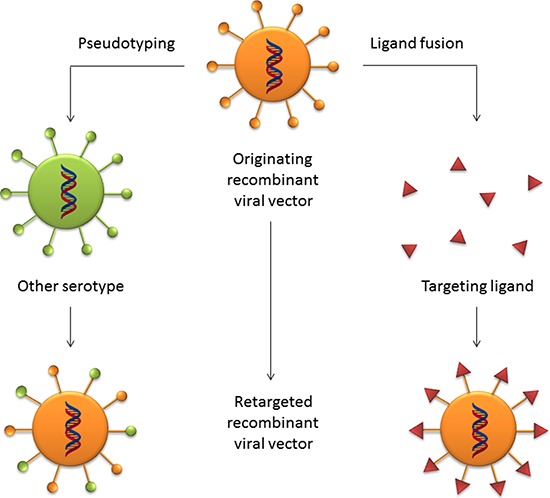
Retargeting strategies for viral vectors The ablation of the natural tropism of viral vectors can be achieved by two means. A common strategy is the pseudotyping technique, by which glycoproteins from other serotypes that exhibit a desired tropism are grafted onto the capsid proteins of the parental viral vector. A ligand with receptor binding proprieties can be fused into the glycoproteins of the parental virus to retarget the vector to a desired group of target cells.

However, the common way of reducing vector tropism towards CAR-expressing cells is to incorporate new functional elements in the viral capsid in order to retarget gene expression to other cell types of therapeutic interest. Incorporation of peptides with ligand proprieties towards specific receptors into the fiber capsid protein has proved to be an efficient approach to target cells expressing low levels of the CAR receptor [73]. Addition of retargeting peptides is made either by direct fusion into the viral fiber protein [74] or by a bifunctional linker such as PEG [74] [70]. Another approach to modify adenoviral vector tropism is capsid pseudotyping. Most of the adenoviral vectors are derived from serotype 5, which displays a high affinity for CAR-expressing cells. Replacing the serotype 5 knob domain of the fiber protein with the serotype 3 knob domain resulted in a fiber chimeric vector that could successfully transduced muscle cells, which normally express low levels of CAR [75]. Likewise, pseudotyping with serotype 35 fiber and penton structures reduced the liver tropism of adenovirus serotype 5 to cells expressing CD46 [76] [77]. These two common re-targeting strategies are schematically depicted in Figure 3.

Another parameter that should be taken in account when designing adenoviral vectors is to choose the appropriate *cis* elements to drive transcription of the therapeutic gene. This is made in a similar way to the non-viral vectors. The use of chimeric CMV/endogenous promoters can give expression levels similar to the ones obtained with CMV promoters, but in a tissue-specific manner [78]. Combining a tissue-specific promoter with a retargeted adenoviral vector could be a better choice to further improve the specificity of therapeutic gene expression [79] [80]. In addition, using elements of the Tet-On regulated system in conjunction with a tissue-specific promoter could be a means of regulating gene transcription in both a spatial and temporal-dependent manner (reviewed in [47]). Also, inclusion of other regulatory elements like the Woodchuck Hepatitis Virus Posttranscriptional Regulatory Element (WPRE) could significantly increase the level of gene expression by elevating the level and stability of nuclear transcripts [81].

Persistence of gene transcription is yet another parameter that could affect the therapeutic response. Adenoviruses harbor DNA genomes that do not integrate into the host genome, and this could be of interest for gene therapy applications where transient gene expression is acceptable. However, in the cases where long term expression of a therapeutic gene product is desired, further optimization of the vector construct is needed. The propriety of retroviruses to integrate into the host genome has gained attention of researchers to develop hybrid adenoretroviral vectors for stable transduction of target cells. Incorporation of the Moloney Murine Leukemia Virus (MoMLV) long terminal repeats (LTR) in the construction of adenoviral vectors, flanking the transgene sequence, resulted in integration of the exogenous DNA into the host genome, without adenoviral sequences [82]. However this poses the risk of insertional mutagenesis. Using shorter LTR sequences could limit this risk without significantly affecting the longevity of the transgene expression, which is higher compared to the adenoviral vectors without retroviral elements [83].

### Replication-competent vectors

Adenoviruses have the ability to infect both dividing and non-dividing cells, where the replication cycle of the virus leads to an increase of viral copies, a process that finally results in destruction of the host cell and release of the newly synthetized viral particles into the surrounding areas. This lysogenic life cycle of adenoviruses has potential therapeutic utility in cases where destruction of “sick” cells is preferred and advantageous rather than correcting the disturbed cellular mechanisms. Cancer has been the main target for virotherapy and oncolytic adenoviral vectors have been designed, in which their viral genome has been modified to selectively replicate in cancerous cells. In addition, suicide genes have been implemented in the construction of oncolytic viruses. These genes have the property to convert a harmless prodrug into a cytotoxic compound, which specifically kills the cell in which the suicide gene is expressed.

The 55-kDa protein encoded by the E1B gene of the viral genome is an inhibitor of the cellular tumor suppressor protein p53. A mutant virus, called ONYX-015, which does not express the 55-kDa protein, has the propriety to selectively replicate in p53-deficient cancers cells, but not in normal cells that express the p53 tumor suppressor. This E1B restricted virus has already been tested in phase II clinical trials of head and neck cancers [84]. Further improvements in terms of efficiency and adenoviral-induced apoptosis have been made by also deleting the E1B 19-kDa protein, which is a potent apoptosis inhibitor. Such E1B 55-kDa/19-kDa-deficient vectors might enhance the therapeutic potential of apoptosis-inducing chemotherapies and radiation therapy [85]. Moreover, deleting the pRb-binding domain of the viral E1A gene generated E1A/E1B double-restricted vectors that have the ability to infect tumor cells with pRb-disrupted pathway [86]. Inclusion of suicide genes, such as herpes simplex virus thymidine kinase (HSVtk) under the control of a tumor specific promoter, in the construction of an E1A/E1B double-restricted vector, can further augment the efficiency and specificity of such vectors, following ganciclovir treatment [87]. Suicide gene complementation has proved to be useful for improving the oncolytic potential of E1B-restricted adenoviral vectors [88].

Insertion of promoters that are upregulated in tumors in oncolytic adenoviral vectors provides an advantage to obtain tumor-selective transcriptional targeting. One possibility towards this end is to replace the promoter that regulates transcription of the *E1A* gene in a E1B55-kDa-defective vector with a tumor-specific promoter, and this arrangement will ensure that replication of the viral vector will be activated only in tumor cells, while normal cells remain unaffected [89] [90]. Further incorporation of genes with tumor suppressor activity in the construction of tumor promoter-regulated oncolytic vectors might prove to be an even more efficient approach to achieve an efficient anti-tumor growth effect [91] [92] [93]. Pseudotyping of the fiber capsid with fiber knob domains of other serotypes, such as serotypes 3 or 35, is yet another way of directing viral oncolysis towards desmoglein-2 expressing [94] or CD46-expressing cancer cells, respectively [95]. Likewise, insertion of targeting peptides in the fiber knob domain can enhance tumor specificity of replication competent adenoviral vectors [96] [97].

Because oncolytic adenoviral vectors harbor viral genes in their construction, systemic administration can lead to an innate and adaptive immune response. Therefore their use is limited to local administration, which is rather inefficient in the case of large tumors where their diffusion is hindered by the extracellular matrix. To overcome this, a biodegradable alginate gel formulation has been shown to improve the anti-tumor efficiency of such vectors to 1.9–2.4 fold compared to naked oncolytic vectors [98]. As in the case of replication-defective vectors, conjugation of oncolytic adenoviral vectors with bioreducible polymers might be a strategy to circumvent the immune response upon systemic administration and to increase the half-life of the vector particles in the blood stream. In one study, a PEG-conjugated ABP (arginine-grafted bioreducible poly(disulfide amine) polymer) oncolytic vector improved the transduction efficiency of tumors vs liver up to 419-fold compared to the naked vector [99]. Such vector formulations can have a great impact in the treatment of metastatic tumors by viral oncolysis.

### Adeno-associated viral vectors

Adeno-associated viruses (AAVs) are characterized by a single-stranded linear genome of 4.7 Kb encapsulated in an icosahedral viral capsid. The viral genome composed of two genes, *Rep* and *Cap*, is flanked by two hairpin palindromic repeat sequences termed inverted terminal repeats (ITRs) of 145 base pairs (bp). In the absence of a helper virus, such as adenovirus or herpes simplex virus (HSV), the AAV genome can persist in the host as an episome, and to a lesser degree it can integrate into the host genome on chromosome 19 at the specific site, called AAVS1, by a process mediated by the Rep protein. In the presence of the helper virus, the AAV undergoes viral genome replication and productive infection. Adeno-associated viruses are capable of infecting both dividing and quiescent cells, which makes them attractive tools for the delivery of therapeutic genes [100].

Recombinant adeno-associated viruses (rAAVs) are generated by replacing the *Rep* and *Cap* genes with a transgene expression cassette. Because of the small sized genome, rAAVs are capable of accommodating small therapeutic genes. For larger genes (>4.7 Kb), strategies have to be implemented to expand the cloning limit imposed by the viral genome. These have included post-transduction concatemerization of the transgene harbored on two different constructs either by *trans*-splicing, or by homologous recombination of two overlapping sequences of the transgene. However, both strategies are limited to the inherent proprieties of the therapeutic gene. A transgene-independent approach has been developed as a potential answer to this limitation, combining features of both the *trans*-splicing and overlapping systems. This dual system uses an engineered highly recombinogenic alkaline phosphatase (AP) sequence inserted in the *trans*-splicing vector system. Upon transfection, the transgene is reconstituted first by homologous recombination of the AP sequence, which results in transgene interrupted by the AP sequence, and subsequent elimination of the AP sequence by splicing [101]. Other strategies have used minimal expression elements in the expression cassette to favor the size limit of the therapeutic gene in a single vector system, bypassing in this way the relatively low expression efficiency commonly seen with dual vector systems [102].

Removal of the viral genes from rAAV vectors for the propose of inserting a transgene expression cassette, renders the production of vectors dependent on a helper construct to provide the Rep and Cap proteins in *trans*. A typical production system for rAAV vectors comprises a vector plasmid (containing the *cis* elements ITR and transgene expression cassette), an AAV helper plasmid and an adenoviral plasmid which provides *in trans* the AAV *Cap* and *Rep* gene products, and some of the complement adenoviral proteins, respectively. All plasmids are co-transfected in a HEK293 producer line that expresses the E1a and E1b adenoviral proteins. A major drawback of this producer platform is scalability of the process and contamination of the rAAV vector batch with adenoviral vectors or replication competent AAVs particles. In one embodiment, these limitations have been partly addressed by substituting the adenoviral vector with an attenuated pseudorabies virus (a herpesvirus of swine) which expresses the Rep and Cap proteins *in trans* [103]. With this system, better titers of rAAV vectors have been obtained compared to the standard method. Others have produced a novel AAV helper vector that includes tissue-specific microRNA binding sites in the *Cap*/*Rep* expression cassette, in order to reduce their expression from rcAAV contaminating vectors in the target tissue, improving in this way the safety profile of the rAAV vector batch [104]. More recent efforts have been directed towards simplified vector producer cell lines, in which a stably transduced HeLaS3 line with a single plasmid containing the vector sequence, as well as the *Cap* and *Rep* viral genes, upon infection with adenovirus yielded high quality rAAV vectors with no detectable rcAAV particles [105]. In addition, introduction of a large intron in the *Cap* expression cassette can limit packaging of the *Cap*-containing sequence in virions, resulting in a vector batch with a reduced immune response towards AAV vector-transduced cells [106].

As in the case of adenoviral vectors the natural tropism of AAV vectors might impede the transfection efficiency when other cell types are targeted. AAV serotype 2 has been the standard delivery adeno-associated system for the past decades. This serotype is known to target heparan sulfate expressing cells, therefore transfection efficiency of AAV2-derived vectors is dependent on the density of these cell surface receptors. Differential tissue tropism of AAV serotypes has been exploited to design AAV2 genome-based vectors with broader cell tropism by the inclusion of capsid proteins from other AAV serotypes [107]. This pseudotyping approach has proved to be efficient in transducing different cell types, such as epithelial airway cells [108], or dermal fibroblasts [109] [110], with potential implications for gene therapy of cystic fibrosis and chronic non-healing wounds, respectively.

Ablation of natural tropism of AAV vectors can also be achieved by mutating the amino acid residues that are implicated in receptor binding. This preference has been shown to reside in lysine residues of the surface-exposed domain, and substitution of even a single lysine (K) with a glutamate resulted in reduced preference for heparin receptors and an increase transduction efficiency in liver parenchymal cells [111]. In addition, serine (S), threonine (T) [112] and tyrosine (Y) [113] residues of the surface-exposed region of the capsid protein have been suggested to be implicated in the cellular trafficking of the AAV particle via the ubiquitination-proteasome degradation pathway. Moreover, tyrosine (Y)-mutated capsid vectors can more efficiently evade immune destruction [114]. Optimal transduction efficiency can further be obtained through combinational mutagenesis of the surface-exposed Y and T residues [115] [116]. Altogether, mutating K/S/T/Y residues can result in vectors with improved transgene expression, suggesting that the surface-exposed region of capsid protein seems to be critical for both vector tropism and cellular trafficking. Such findings are also supported by a previous study, in which random mutagenesis in this region changed the tropism and transduction efficiency of capsid mutated AAV vectors [117].

Insertion of peptide ligands at specific sites in the capsid protein is yet another approach to re-target the AAVs vectors to a particular cell type. However, when designing such capsid-modified vectors, one must take into account stearic conflicts with the capsid proteins, that might affect proper assembly of the viral particle and hence transfection efficiency. In this regard, selecting peptides from peptide libraries displayed on AAV capsids might prove to be advantageous over phage displayed libraries [118]. In addition to peptide ligands, other types of moieties can be attached to viral capsids. Fusing affinity tags to re-targeted peptide-modified vectors permits relatively easy production and purification of AAV vectors with modified tropism for gene therapy applications [119].

Upon transduction of the target cell, the AAV particle escapes the endosome and translocate to the nucleus, where the viral capsid is uncoated and the single stranded DNA undergoes double strand synthesis. This process has been shown to be a limiting step in efficient transgene expression, which is subjected to cellular factors (FKBP52) that prevent second strand synthesis by interacting with the ITR of the AAV genome [120]. Cumbersome improvements have been made to increase the efficiency of single-stranded AAV vectors (ssAAVs). The development of self-complementary AAV vectors (scAAVs) harboring natural inhibitors of FKBP52 (TC-PTP and PP5) has been shown to increase the transduction efficiency of single-stranded AAV vectors [121]. Such system would imply the use of a quadruple-plasmid transfection system for generating a mixed population of ssAAV and scAAV vectors [122] [123]. A further improvement to this method was to insert the transgene expression cassette into a scAAV vector, thereby eliminating the need for the scAAV-TC-PTP/PP5 vector. Mutating the tyrosine residues from the surface-exposed domain of the viral capsid can increase transgene expression efficiency of scAAV vectors [124] [125].

Design of the therapeutic gene expression cassette is also of major importance when designing AAV vectors. Choosing the appropriate promoter to drive the expression of the transgene could have an impact on the efficiency of the therapeutic response. Like in other gene therapy delivery systems, the use of disease or tissue-specific promoters has been preferred over the CMV promoter in order to obtain targeted transcription of the therapeutic gene [126] [127] [128]. Expression levels of the therapeutic gene can further be improved by fusing the CMV enhancer into the tissue-specific promoter sequence, resulting in hybrid promoters [129] [130]. In clinical applications where controllable therapeutic gene expression is required, such as for cytokine expression, using inducible promoters could be a preferred alternative to other types of promoters. The tetracycline/doxycycline regulated Tet-On system has expended its applicability also to AAV vectors. However, because of limited cloning capacity, modifications have been made to adapt this inducible system to AAV vectors. In this regard, a bidirectional TRE promoter has been designed to drive both transcription of rtTA and the transgene [131] [132]. In the absence of the inducer (tetracycline/doxycycline), the expression of the transgene is halted to avoid any undesired side effects.

The sustainability of therapeutic gene expression is limited to non-dividing cells, where the presence of the AAV vector as an episome is sufficient for stable expression. However in the case of dividing cells, the therapeutic gene is lost along with the AAV vector genome with each successive cell cycle. Therefore integrating vectors would be desirable for such cases. The wild-type AAV, in the absence of a helper virus can integrate at a specific site on the human chromosome 19, a process mediated by the Rep protein [100]. However recombinant AAV vectors tend to randomly integrate in the host genome, showing a preference for genes, regulatory sequences, ribosomal DNA sequences and palindromic sequences. Even such insertions have been associated with proto-oncogenes sites, and some vectors are more prone to induce genotoxic effects than others by an unknown mechanism [133]. To increase the safety of integrating vectors, site-specific homologous sequences could be included in the construction of such vectors. One study has shown that flanking the transgene expression cassette by a 1 Kb 28S rDNA sequence resulted in a 30 fold higher integration frequency than in controls, which occurred at the 28S rDNA genomic locus [126]. Homologous recombination integrating vectors could be a promising approach to maintain a prolonged expression of the therapeutic gene in a population of dividing cells, which reduces the risk for insertional mutagenesis.

### Retroviral vectors

Another important class of vectors for gene therapy applications is derived from retroviral viruses. In these pathogens, a diploid (+) RNA genome is transcribed into a DNA intermediate upon infection. This viral DNA integrates into the host genome and functions as a provirus. Simple retroviruses, like onco-retroviruses, have a genome composed of four genes (*gag, pol, pro, env*), while more complex retroviruses, like lentiviruses, have an additional set of accessory genes. Two long terminal repeats (LTRs), which enclose the viral genes, represent together with the primer binding site (PBS), polypurine tract (PPT) and the packaging signal (ψ), the *cis* viral elements, while the *trans* elements are represented by the genes that encode the viral proteins. Each LTR has an integrase attachment site (*att*), by which the integrase mediates the integration of the viral genome into the host genome [134].

The potential that retroviral vectors might have for the treatment of human diseases was demonstrated in early clinical trials on patients suffering from X-linked severe combined immunodeficiency (SCID-X1). After treatment, the symptoms of most of the patients improved; however a few developed a lymphoproliferative disease, due to insertional mutagenesis [3] [135]. Therefore the safety and efficacy of these vectors is of utmost importance and various attempts have been made to improve them.

The important matters to consider when designing a vector include limiting of insertional mutagenesis, infectivity of cell types, internal promoter selection and how vector elements affect virus titer and transduction. These matters, which are important for all types of retrovirus vectors, are discussed below.

In order to limit oncogene activation, self-inactivating (SIN) vectors have been developed by deleting the enhancer/promoter sequences of the U3 region of the 3′LTR. Upon transduction of target cells, the 3′ LTR is duplicated during reverse-transcription, therefore the provirus will lack any endogenous promoter activity. This arrangement also favors the internal promoter of the transgene expression cassette compared to conventional vectors, in which promoter interference between the LTR promoter and the internal promoter can occur. However, residual U3 promoter activity has been detected in many SIN vectors and additional deletions in this region are necessary to attenuate viral promoter activity [136]. In addition, read-through transcription has been observed in SIN vectors, which has the potential of activation of nearby genes. Inclusion of polyadenylation enhancer sequences in the U3 region might prove to be sufficient to generate proper terminated transcripts [137].

The capsid of retroviral vectors has been extensively subjected to pseudotyping with different viral glycoproteins in order to increase the infectivity spectrum of these vectors, and hence their applicability for gene therapy protocols. The vesicular stomatitis virus G glycoprotein (VGV-G) has been a primary means to broaden cellular tropism of retroviral vectors; however such pseudotyped vectors present an associated toxicity and are prone to inactivation by human serum. Directed evolution of VGV-G has proven to be a useful tool to overcome the limitations of VGV-G pseudotyped onco-retroviral vectors [138]. Still, numerous efforts have been directed to other types of viral glycoproteins that could alter the restrictive tropism of retroviruses towards CD4+ cells. Glycoproteins derived from other retroviruses like gibbon ape leukemia virus (GaLV) and murine leukemia virus MLV (10A1) have been shown to be valuable tools to redirect retroviral vectors towards CD34-positive cells [139]. These types of cells can also be successfully transduced by retroviral vectors pseudotyped with the feline endogenous virus RT114 glycoprotein [140] [141] and cocal vesiculovirus glycoprotein [142]. Cells of the immune system, such as lymphocytes, have been targeted with vectors pseudotyped with glycoproteins derived from Measles virus [143] or Tupaia paramyxovirus [144]. Such capsid chimeric vectors might have a relevant clinical importance for lymphocyte-gene therapy and immunotherapy. Other efforts have been directed to re-target retroviral vectors towards airway epithelial cells, which might have an impact in treatment options for lung diseases such as cystic fibrosis. The property of Sendai virus (SeV) to bind sialic acid and cholesterol receptors has been exploited to efficiently transduce epithelial cells by pseudotyping the simian immunodeficiency virus (SIV) capsid with the HN and F envelope proteins of SeV [145] [146]. The attachment and fusion proteins of Nipah virus can be used to specifically transduce endothelial cells [147]. In addition, the xenotropic and polytropic retrovirus receptor 1 (XPR1) expressing cells, such as pancreas, kidney, heart and hematopoietic cells can be successfully targeted with retroviral vectors pseudotyped with the murine leukemia virus-related virus (XMRV) Env protein [148]. Therefore, when one wants to design a LV vector that is intended to transduce a specific cell type, searching for other viral glycoproteins that have a receptor on that particular cell type, is the first step in the pseudotyping method of altering the vectors' natural tropism. Likewise, ligands with receptor specificity could also be fused to pseudotyped retroviral vectors to improve transduction of target cells in a cell-type specific manner [149].

The internal promoter that drives therapeutic gene expression is also a key factor that might influence the therapeutic effect of the SIN vector. Designing vectors with a composite inducible/tissue-specific promoter could be a means to improve both the efficiency and specificity of the retroviral vector [150] rather than using a constitutive unspecific promoter. Other elements to include in designing the expression cassette might be represented by internal ribosome entry sites (IRES) that gives the possibility to express two different proteins from the same transcript [151]. The bicistronic arrangement could address the deficiency of the targeted disease at multiple levels.

The type of promoter that drives the expression of the therapeutic transgene is yet another element to be evaluated in the vector backbone. Several attempts have been made to determine if an endogenous or exogenous promoter is to be preferred to obtain satisfactory therapeutic expression levels. In a recent study, CMV displays better expression levels of the transgene compared to endogenous promoters, such as human elongation factor-1 alpha (EF1α) and phosphoglycerate kinase (PGK), two commonly used constitutive non-viral promoters [152]. However, the transduction specificity in such cases would be determined by viral capsid tropism. A step forward can be made using promoters that are expressed in a particular cell type and this can lead to expression of the therapeutic gene in a cell-specific manner. For example the human telomerase reverse transcriptase (hTERT) promoter, whose expression levels are elevated in cancer cells but not in normal cells, can drive expression of a suicide gene to specifically target and kill cancer cells [153]. In addition, a tissue-specific promoter can be used to obtain long term therapeutic gene expression [154], as constitutive viral promoters are prone to inactivation. The genomic insertion site could be an important factor that leads to promoter silencing. A potential promoter candidate has been identified as the ubiquitous chromatin-opening element (UCOE) that is able to drive stable and robust gene expression levels independent of the insertion site [155]. Moreover, insertion of post-transcriptional regulatory sequences, like the woodchuck hepatitis virus posttranscriptional regulatory element (WPRE), downstream of the transgene, can further improve therapeutic transgene expression levels driven by tissue-specific promoters [156].

In some instances, temporal control of transgene expression is desirable in addition to cell type-specific expression, and different strategies have been developed toward this end. The Tet-ON system is a useful means of obtaining drug inducible transgene expression, as in the case of other types of vectors (see above). In one study, a LV doxycycline-inducible vector has been designed by placing the TetR under the control of the spleen focus forming virus (SFFV) and the transgene under the control of the regulated CMV-TetO promoter [157]. Bi-directional Tet-inducible promoters have also been previously described to regulate the simultaneous expression of two genes from the same construct, or to correlate the activity of a transgene with that of a reporter gene, without the constraints of IRES bicistronic arrangements [158]. Others have used miR target sequences to tag the repressor transcriptional unit to create switch LV vectors. The transgene is ON only when the miR is active; therefore such a system is cell-type induced by a specific miR [159].

Vector elements can significantly affect virus titer and transduction. The vector elements should thus be selected with care. Posttranscriptional regulatory elements, such as the one derived from woodchuck hepatitis virus (WPRE), has been shown to enhance both transduction and vector production, especially when two copies of this element have been inserted 3′ of the transgene [160]. However, the WRPE can pose a safety risk regarding associated hepatocellular carcinoma, and mutant variants have been developed to reduce this risk [161]. Other studies have been directed to the central polypurine tract (cPPT) to improve retroviral vector transduction efficiency, resulting in an increased copy number of integrated proviral DNA [162]. Such vectors, harboring the cPPT, can further be improved in terms of transduction efficiency by adding a matrix attachment region (MAR) of the immunoglobulin-k into the vector backbone [163]. In addition, combination of both WRPE and cPPT were tested in order to increase retroviral vector transduction efficiency [164].

The 1.2-kb chicken β-globin locus HS4 (cHS4) insulator element, which is commonly used to reduce the insertional mutagenic potential of retroviral vectors, can also affect both titers and efficiency of such vectors. Generation of a smaller 0.25kb core element of cHS4 can save vector titers, while still maintaining the transduction efficiency of retroviral vectors harboring this truncated form of the cHS4 insulator [165].

#### Onco-retroviral vectors

The majority of vectors derived from onco-retroviral viruses are based on the Moloney murine leukemia virus (MoMLV). In these vectors, the *trans* viral genes have been deleted from their construction and render them dependent on a helper system to provide the viral proteins in *trans* as in other types of virus-based vectors (see above). MoMLV-derived vectors preserve in their construction the *cis*-acting elements necessary for packaging and expression, while the transgene therapeutic expression cassette replaces the viral genes [166].

Integration of retroviruses has been shown to be directed towards proto-oncogenes rather than a random integration [167], as previously reported in a gene therapy clinical trial [3]. Moreover, MoMLV integrations could also induce genomic instability, which promotes neoplastic progression [168]. Activation of oncogenes is most likely a result of the strong promoter activity of the U3 region of LTRs. Indeed, the MoMLV shows an integration bias towards the start of transcriptional units [169] [170] and this has been proposed to be determined by the host transcriptional factors interaction with the enhancer elements in the LTR sequence, which synergize with the integration of the provirus in transcription units [171] near regulatory elements like enhancers and promoters of active chromatin regions [172] [173].

#### Lentivirus vectors

A more preferred type of vector of the retrovirus class is derived from lentiviruses (LV), especially HIV-1. In contrast to onco-retroviruses, LVs are capable of transducing both dividing and non-dividing cells, which broadens the applicability of this type of viruses for gene therapy applications. In addition, they present a better safety profile regarding activation of proto-oncogenes upon insertion into the host genome. Their preference seems to be biased towards transcriptional units rather than 5′ regulatory sequences, as in the case of MoMLV [174] [175]. However, new lines of evidence suggest a carcinogenic effect of LV integration in the host genome [176], although such oncogenic potential might be more limited compared to MoMLV. Development of integration-deficient LV vectors could be the answer to overcome this relative limitation [177] [178], but would compromise the sustainability of transgene expression obtained with integrating vectors.

The construction of vectors derived from LVs is largely made in the same way as for onco-retroviral vectors. However, LV vectors preserve the *gag* gene in a non-functional state, as well as the rev responsive element (RRE), by which the rev protein mediates the nuclear export of viral ARN [179]. The expression transgene cassette replaces the viral gene, therefore the viral genes are provided from a first generation helper construct. Because the viral accessory genes, which are responsible for pathogenicity, are not necessary for gene transfer, they have been deleted from the helper construct, and a second generation helper construct has been obtained. A further improvement to this helper system has been made by providing the regulatory Rev protein from a separate construct; therefore co-transfection of the *rev* construct with the *gag-pol* construct is necessary for production of vector particles [180] [181]. This third generation helper system lowers the risk of recombination of the helper construct with the viral vector, which results in lower titers of replication-competent vector particles. Stable transduced packaging cell lines represent a next step to reduce the risk of replication-competent vectors. An inducible packaging system has been described, in which the viral genes stably transduced in a cell line are under the control of a minimal Tet(o) promoter. The presence of tetracycline ensures a tight control of expression of the viral genes [182]. Different stable transduced packaging cell lines have recently been described as a feasible method to obtain high titers of clinical grade retroviral vectors with improved safety profile [183] [184].

Besides the viral capsid, the vector backbone has also gained the attention of researchers as a means of improving the safety and efficiency of gene delivery. The first step to improve the safety profile of LV vectors was to delete the U3 region of the 3′ LTR, resulting in self-inactivating vectors, in which the viral transcription capability is lost upon transduction, minimizing the promoter interference phenomenon and the risk of activating nearby genes at the site of genomic integration. A second step would be inclusion of insulators, which are genomic DNA sequences that are capable of preventing interaction between the integrating vector and the adjacent regulatory genomic sequences. They also prevent transgene silencing by the chromosomal position effect [185]. The chicken hypersensitivity site 4 insulator (cHS4) has been the standard for improving transgene expression in several studies [186] [187] [188] [189]. This type of insulator enhances vector titers compared to LV vectors containing other types of insulators such as the locus control region [187]. Moreover, the cHS4 insulator has been modified by fusing it to the scaffold attachment region (SAR) element, resulting in a chimeric insulator that further improves vector titers and transgene expression of the LV vector [190]. Besides cHS4, other types of genomic insulators have been identified and validated as potential elements to include in LV vector design to improve their efficiency and to reduce their oncogenic potential [191].

#### Foamy virus vectors

An emerging type of viral vector is derived from foamy viruses (FV), a spumavirus subfamily of *retroviridae*, which are capable of transducing a variety of both dividing and non-dividing cells, such as hematopoietic cells, lymphoid cells, epithelial cells and fibroblasts [192] [193] [194], and also neuronal cells [195] [196], which makes them attractive for gene therapy applications of the nervous system. Although the FV receptor seems to be present on cells derived from different vertebrate species [197], it has only recently been identified as being a heparin sulfate receptor [198]. In addition to their broad tropism, they offer several other advantages over conventional retroviral vectors, such as accommodation of large transgene expression cassettes, lack of pathogenicity and minimal genotoxicity, even when inserted in the vicinity of host chromosomal genes [199] [200].

Although replication-competent recombinant FV vectors have been described [201], the helper-dependent system is the preferred structural conformation for generating recombinant FV vectors. In this type of vector, the presence of *Bel-1* trans-activator protein gene could lead to replication competent vectors. A chimeric CMV-LTR promoter has been generated to drive transcription of both helper and vector genomes, limiting the requirement for Bel-1 protein [202]. Stably transduced packaging cells lines have also been generated to increase vector titers, bringing LV vectors a step closer to clinical applications [203]. To this end, generation of SIN-FV vectors with minimal core elements further increased the safety of these vectors, while the transgene has been placed under the control of an endogenous promoter, such as the ubiquitin C promoter [204].

### Other viral-derived vectors

The search for safe and more efficient gene delivery vectors based on viruses has led researchers to investigate alternatives to the above mentioned virus types. Among these, the *Herpes simplex* virus (HSV) and poxviruses (PV), like vaccinia virus, have undergone gene therapy clinical evaluation [4]. HSV has the ability to efficiently infect cells of the central and peripheral nervous systems. Generation of genetically engineered HSV has gained attention as a potential means of treating neuronal diseases. The viral genome of HSV is comprised of 152 kb double-stranded DNA that harbors two sets of genes. The immediate early genes are necessary for life cycle initiation and expression of the remaining viral functions, while the late viral gene set is responsible for the lytic cycle. Most of the viral genome is dispensable; therefore HSV vectors are capable of accommodating large transgenes or multiple genes. Two immediate early genes have been deleted to generate replication-defective HSV vectors. These genes, termed *ICP4* and *ICP27*, are essential for entering the lytic cycle and viral DNA replication. *Trans*-complementing cell lines for ICP4 and ICP27 proteins have been developed to generate HSV vectors. However, the vector titers are affected by delayed expression between ICP4 and ICP27 genes, with the latter being favored upon vector infection. Improvements to this system have been made by placing the ICP4 gene under the control of the ICP0 promoter, which is enriched in recognition sequences for the viral VP16 transactivator [205]. This arrangement ensures that ICP4 and ICP27 gene expression is synchronized, and hence higher HSV vector titers are obtained.

Optimization of transgene expression has also been evaluated for other viral vectors. Tissue-specific promoters restrict HSV vector transgene expression to a particular neuronal cell type, and sustain prolonged expression levels [206] [207]. Also, the Tet-inducible system has been successfully used to control transgene expression in a tetracycline-dependent manner in replication-defective HSV vectors [208] [209]. In addition to non-proliferative cells, like neuronal cells, HSV vectors have also been engineered to transduce dividing cells like glial cells of the nervous system. This has been achieved by fusing *cis* responsive elements of cell cycle progression factors to a tissue-specific promoter, restricting transgene expression to glial cells [210]. These cell cycle-dependent and glial-specific HSV vectors could be very useful tools for targeting brain tumors like glioblastoma. Locally administered recombinant HSV vectors showed activation of the immune system, without affecting surrounding cells [211]. Beside the promoter, other elements of the vector backbone could also affect the level and sustainability of transgene expression. The U_L_ 13,46,47 gene proteins have been shown to influence transgene expression, and mutating these viral proteins should be considered when a prolonged expression of the therapeutic gene is desired [212].

The HSV lytic cycle might be exploited as a mean of eradicating cancer cells. Brain tumors, such as gliomas, might be the first to benefit from such treatment. Towards this end, a double mutated vector G207, containing a deletion of the *γ34.5* gene responsible for neurovirulence and a *LacZ* gene insertion disrupting the *ICP6* gene encoding the large unit of the ribonucleotide reductase, has been generated to replicate and kill cancer cells that are able to compensate for the non-functional genes [213]. Moreover, this double-mutated HSV vector has attenuated pathogenicity, along with effective killing of cancers cells when compared to the wild-type virus. To improve the anti-tumor efficacy of the oncolytic HSV vectors, further mutations have been employed. Triple-mutated oncolytic vectors were generated by further deleting the non-essential viral gene α47, resulting in enhanced replication within tumor cells [214] [215]. *γ34.5*-deleted variants have also been tested, in order to identify the most efficient mutated vector that is both safe and efficient in targeting and eradicating various brain tumors [216]. Other efforts to improve the anti-tumor efficiency have been directed to creating membrane-enhanced fusogenic vectors by inserting a hyperfusogenic glycoprotein into a mutated HSV vector. Such vectors proved their efficiency against metastatic cancers [217] [218]. Furthermore, Takaota and colleagues, and Meshii and colleagues described mutated HSV vectors that combine the tumor-specific replication of the *γ34.5*-defective phenotype with the highly fusogenic characteristic of the HSV HF10 strain [219] [220].

Targeted replication of oncolytic HSV vectors has also been the subject of experimentation to enhance tumor specificity. Insertion of antibodies to tumor-specific receptors into the virus envelope glycoproteins is one way of improving the safety of systemically administrated oncolytic HSV vectors [221] [222]. Fu and colleagues [223] incorporated specific miR sequences to essential viral genes under a liver specific promoter to increase the targeting of cancer cells. This arrangement ensured that viral proteins are expressed in cancer cells which are deficient in the specific miR, while in the normal cell, which expresses the miR, viral replication is prohibited by degradation of the viral gene transcripts.

Other types of viruses such as members of the *Poxvirus* family (PV) have proved their efficacy as effective transgene delivery vectors [224] [225] [226] [227] [228]. Their efficiency has also been evaluated in gene therapy trials [4]. Their safety profile with regard to activation of the immune response seems to vary among different strains, vaccinia virus Ankara strain being the most promising to elicit a lower immune response towards its own viral antigens [229]. As for the case of HSV vectors, oncolytic poxvirus mutants have also been generated by deleting the thymidine kinase gene (TK). Such replication-competent PV vectors show preference for tumor cells after systemic administration [230]. Oncolytic PV vectors harboring suicide genes outperformed equivalent oncolytic adenoviral vectors in inhibition of tumor growth [231]. Furthermore, vaccinia PVs are capable of eliciting an anti-tumor effect on hypoxic tumors, which makes them attractive tools for targeting pancreatic tumors or other hypoxic tumors [232]. Dissemination of the oncolytic PVs in the host after tumor lysis is important for targeting metastatic tumors. Towards this end, an *A34R* gene-mutant oncolytic variant is a promising vector for such clinical applications, with improved spreading of virus progeny and resistance to complement neutralization and poxvirus-specific antibodies [233]. Other improvements to oncolytic poxviruses have included “arming” these vectors with immunostimulatory cytokines [234] or cell surface tumor-specific antigens [235].

HSV oncolytic vectors, as well as PV oncolytic vectors, have proved their efficiency in clinical settings. Oncovex^GM-CSF^ is an *ICP34.7/ICP47*-deleted vector in which the granulocyte macrophage colony-stimulating factor gene (GM-CSF) was inserted in the *ICP34.7* locus. Expression of this factor stimulates maturation of antigen-presenting cells, such as dendritic cells and the cellular immune response. Therefore, following administration at the primary tumor site and local oncolysis, a secondary humoral tumor-specific immunity develops which could target distant metastatic tumors. In a randomized Phase III clinical trial on melanoma cancer patients, OncoVEX^GM-CSF^ administration leads to regression of both injected and non-injected lesions [236].

As for PV oncolytic vectors, Phase II clinical trial liver cancer patients presented significantly better overall survival rates after administration of an improved PV oncolytic vector, named JX-594 [237]. This vector also displays the *GM-CSF* gene, which is inserted in the thymidine kinase (TK) locus, rendering the vector tumor-selective.

These clinical studies highlight the feasibility of oncolytic immunotherapies as a potential means of treating of both primary and disseminated tumors, and acquisition of a subject-specific anti-tumor immunity.

### Hybrid vectors

What we have seen for the past decades is a divergent evolution of vectors derived from the “primordial” vectors, which have been used in the early beginning of gene therapy. But perhaps now will be a new beginning for a convergent evolution of the existing gene therapy vectors. This vision has started to take shape in the development of hybrid vectors that combine features of both non-viral and viral vectors. One possible hybrid vector could comprise a viral vector which is conjugated with a synthetic biocompatible polymer, resulting in ablation of the natural tropism of the native virus and enhanced transduction towards cells that do not express their cognate receptors [238]. However, such vectors could still elicit a potential immunologic response to the viral constituents of the vector. In addition, production of such vectors implies both production in helper cell lines and cumbersome purification methods of the recombinant vector, and subsequent conjugation with the polymer.

Another advance in the field of hybrid vectors is the development of virus-like particles or shortly VLPs (reviewed in [239] [240]). These particles have been described as self-assembling recombinant viral protein structures, which resemble the parental viral particle in morphology and infectivity, but are devoid of the viral genome. This design would limit the commonly seen issues associated with viral vectors, and the production procedure, which permits expression of the recombinant viral proteins in heterologous expression systems like *E. coli*. VPLs have primarily been developed for vaccine applications, but they are also capable of successfully transporting exogenous genetic material, as previously shown [241] [242] [243]. The efficiency of VLPs can further be enhanced by complexation with synthetically materials, such as liposomes [244] or cationic polymers, such as chitosan [245].

Another relatively new and emerging class of promising gene delivery vectors is represented by a hybrid AAV viral genome and the M13 bacteriophage genome (AAVP), which is encapsulated in a bacteriophage capsid [246]. This type of vector offers several advantages over other types of gene delivery vectors, because the bacteriophage does not have natural cognate receptors in mammalian and human cells, and by engineering the phage capsid with a receptor-specific ligand, a highly targeted gene delivery vector can be obtained. The presence of the AAV genome improves the metabolism of the transgene and offers sustained expression. In addition, high titer AAVP particles can be obtained in bacteria in only three days [246], which gives the possibility to extrapolate the production procedure at a large scale, without the expenses of using helper cells lines, as in the case of viral vectors. It is worth to note that bacteriophages are safe for systemic administration in humans, and even in children, as they have been previously used as antibiotic therapy. A more detailed description of bacteriophage-derived vectors can be found in two recent published reviews ([247] [248]).

Despite these encouraging characteristics of AAVP vectors, there are also some shortcomings that arise with the use of such vectors. First of all, an AAVP vector functionalized with a targeting peptide, such as RDG which binds the α_5_β_3_ integrin, is internalized in a clathrin-mediated manner and subjected to the endo-lysosomal pathway, and this can limit the transduction efficiency of AAVP vectors [249]. In addition, the phage capsid proteins are subjected to polyubiquitination and proteasome degradation, and such metabolism of the capsid would be a major drawback for anti-cancer therapy, in which the proteasomal pathway is over-expressed [250].

## SUMMARY AND FUTURE PROSPECTIVE FOR GENE THERAPY VECTORS

As described in the previous pages, numerous efforts have been made to move this innovating potential therapeutic approach from the bench to the bedside. However, the fact that a large array of gene therapy vectors have been developed and only one product is approved for clinical use, suggests that delivering a genetic therapeutic product to a specific group of target cells without local or systemic adverse events is more complex than previous thought, and that future research is needed to overcome the limitations that gene therapy vectors impose.

Table 1 are summarizes some of the main advantages and disadvantages of commonly used gene therapy vectors intended for clinical applications.

**Table 1:** Basic characteristics of conventional gene therapy vectors

Vector type	Description	Transduction efficiency	Specificity	Clinical applications*	Safety profile	Production	References
Non-viral	Naked genetic material or complexed with a chemical compound	Low	Low	Cancer, cardiovascular diseases, cystic fibrosis	Relatively good	Relatively easy	6, 7, 8, 9, 14, 30
Adenoviral	Double-stranded DNA viruses in which the therapeutic gene replaces the structural genes (oncolytic vectors make an exception)	High	CAR receptors	Cancer, cardiovascular diseases, neurodegenerative disorders, diabetes, metabolic diseases, cystic fibrosis, angina pectoris, OTC deficiency	Highly immunogenic	Difficult	47, 60, 61, 62, 63, 64, 65
Adeno-associated virus (AAV)	Single-stranded DNA viruses in which the therapeutic gene replaces the structural genes	High	Heparan sulfate receptors (wide tropism)	Hemophilia, neurodegenerative disorders, retinal diseases, muscular dystrophies, cancer, cardiovascular diseases, metabolic diseases, hepatitis C	Relatively good	Difficult	100, 103, 107
Retro-viral	Single-stranded RNA viruses in which the therapeutic gene replaces the structural genes	High	CD4+ receptors	Cancer, SCID, inherited anemia, retroviridae infections	Potential genotoxic effects	Difficult	134, 135, 138, 139, 166

www.genetherapy.net; https://clinicaltrials.gov/

Non-viral gene therapy vectors, in contrast to viral vectors, have low transduction efficiency, and their specificity is limited and dependent on functional groups attached to the delivery complex. This process implies cumbersome modifications to their structure in order to be able to achieve a stable and efficient gene therapeutic delivering vector. However, non-viral vectors display a relatively superior safety profile and their chemical nature allows production on a large scale, compared to viral vectors, which are largely dependent on helper cells lines to obtain infectious particles. In addition, obtaining good titers of high quality viral vectors is rather complicated and expensive.

The efficiency of transduction of viral vectors is by far superior to non-viral approaches. However, their specificity is directed to cells that express their cognate receptors for internalization of the viral particle. The immunogenic and genotoxic effect, in the case of integrative vectors, is one of the major drawbacks which limit the clinical applicability of viral vectors.

Therefore, an “ideal” gene therapy vector should have high transduction efficiency, but limited to a group of target cells. In addition, such a vector should display a high safety profile which can allow it to be systemically administrated without any cyto-/geno-toxic adverse events. Another aspect to be taken into consideration is scaling-up the production of such vectors, which should be easy and relatively inexpensive.

These characteristics of an “ideal” gene therapy vector might suggest that a compromise between non-viral and viral vectors could be one solution to solve these yet unsolved limitations that we currently experience with the use of gene therapy vectors. In this regard, “convergent” hybrid vectors might represent promising tools for safe and efficient transfer of therapeutic genes, moving gene therapy a step closer to the clinic. Among these, the viral/phage hybrid vectors could bring a contribution towards this end. Finding alternative means for endocytosis/proteasomal-independent intracellular trafficking could further improve the transduction efficiency for these hybrid vectors.

Designing a hybrid viral/phage vector with oncolytic potential would be an appealing research direction for anti-cancer therapies. Such hybrid viral/phage vector would be comprised of the phage capsid on which a targeted ligand is expressed to improve the targeting specificity of the vector. The genetic material would contain both phage elements for assembly into phage particles, and elements of an oncolytic viral genome. Upon transduction of the cancer cells, the vector is internalized and the oncolytic viral genes are transcribed into structural and functional proteins for assemble in oncolytic viral particles. These virions would undergo viral oncolysis and cell lysis of cancer cells. A further step to improve organ specificity and tumor targeting could be achieved by incorporating miR recognition sequences to the viral genes, which are placed under the control of a tissue specific promoter. This would ensure that the viral genes are transcribed only in a particular cell type where the tissue-specific promoter is expressed, and only in cancer cells that are deficient in that specific miR [223].

However treatment of distant metastatic tumor still poses a challenge for anti-cancer genetic therapies. One strategy, which has been previously described [236], is to insert the granulocyte macrophage colony-stimulating factor gene (GM-CSF) into the vector backbone. GM-CSF would elicit a secondary tumor-specific immune response subsequent to viral oncolysis that could treat distant metastatic sites.

In summary, a hybrid viral/phage that expresses a tissue-specific ligand on the phage capsid would be an appealing alternative to the viral capsid-based vector for targeting specific organs and to evade the innate and adaptive immune response upon systemic administration. In addition, a challenge would be to design a hybrid viral/phage with oncolytic potential that would target tumor cells either by systemic administration or by producing a secondary tumor-specific immunity upon local administration.
